# A cross-sectional study of the relationships between different components of sarcopenia and brachial ankle pulse wave velocity in community-dwelling elderly

**DOI:** 10.1186/s12877-020-01525-8

**Published:** 2020-03-30

**Authors:** Yu-Dong Rong, Ai-Lin Bian, Hui-Ying Hu, Yue Ma, Xin-Zi Zhou

**Affiliations:** 1 Health Management Centre, Tianjin First Central Hospital, No. 24 of Fukang Road, Nankai District, Tianjin, 300192 China; 2 Department of Geratology, Tianjin First Central Hospital, No. 24 of Fukang Road, Nankai District, Tianjin, 300192 China; 3 Department of Geratology, Yanda International Hospital, Langfang, Hebei, 065201 China

**Keywords:** Elderly, Sarcopenia, Arterial stiffness, Brachial ankle pulse wave velocity

## Abstract

**Background:**

Previous studies have just found skeletal muscle mass decline is associated with arterial stiffness, but it is unclear whether muscle strength and physical performance as important compositions of sarcopenia are associated with arterial stiffness. The aim of this study was to investigate the relationship between sarcopenia, the components of sarcopenia and arterial stiffness among elderly in the community.

**Methods:**

This study enrolled 450 elderly people who received general medical examinations in Tianjin First Center Hospital. Each of the subjects was greater than 65 years old, including 266 male and 184 female subjects. Based on the diagnostic criteria for sarcopenia in older people developed by the Asian Working Group for Sarcopenia (AWGS), 89 subjects were separated into the sarcopenia group. The living habits, disease status, general status and laboratory examinations of all subjects were collected. The body composition (including appendicular skeletal muscle mass and visceral fat area (VFA) of each participant) was measured by bioimpedance analysis. HS, usual gait speed (GS), and brachial ankle pulse wave velocity (baPWV) were measured.

**Results:**

Sarcopenia subjects had higher baPWV, nutrition risk and lower appendicular skeletal muscle index (ASMI), Handgrip strength (HS), GS, body mass index (BMI), triacylglycerol (TG), serum albumin (ALB) and creatinine (Cr) than did non-sarcopenia subjects; Sarcopenia subjects also had higher visceral fat area (VFA) than did non-sarcopenia subjects (*p* < 0.05). ASMI and HS were negatively associated with baPWV (t = − 5.807, *p* = 0.000 and t = − 3.085, *p* = 0.002), but the relationship between baPWV and GS was not statistically significant (t = − 0.862, *p* = 0.389) by multivariable linear regression. After adjusting for confounders, a multivariate logistic regression analysis revealed that sarcopenia was related with age, BMI, sports and baPWV in community dwelling elderly.

**Conclusions:**

ASMI and HS were negatively associated with baPWV in community dwelling elderly in China; and baPWV was a risk factor of sarcopenia.

## Background

Sarcopenia has been recognized as a muscle disease with low muscle strength, low muscle quantity or quality and low physical performance [1]. Sarcopenia is common among eldly and easily neglected, yet it can cause many adverse outcomes including increased risk of falls and fractures, prolonged time in bed, increased risk of infection, and increased rates of cardiovascular disease with a poor prognosis, unfavorable metabolic effects, disability and mortality in older people [2–4].

The underlying pathogenesis of sarcopenia including low physical activity, increases in oxidative stress and insulin resistance, changes in aging-related hormones and increased levels of inflammatory factors, which have been showed to promote alterations in arterial stiffness and atherosclerosis. Therefore, there is believed to be a relationship between sarcopenia and arterial stiffness. Several studies have found that skeletal muscle mass decline is associated with arterial stiffness [5, 6]. But sarcopenia is not only determined by muscle mass, but also by muscle strength and physical performance. Furthermore, low muscle strength is now considered overtaking the role of low muscle mass as a principal determinant for sarcopenia [1]. However, the previous studies did not involve muscle strength and physical performance [5, 6]. In fact it is unclear whether sarcopenia (according to the diagnostic criteria for sarcopenia by AWGS in 2014 [7]) and the important compositions of sarcopenia-muscle strength and physical performance are associated with arterial stiffness. The present study was therefore designed to investigate the relationship between sarcopenia, the components of sarcopenia and arterial stiffness according to the Consensus Report of the Asian Working Group in 2014 in Chinese community dwelling elderly.

## Methods

### Research subjects

For the current study, we recruited participants from the people who attended general medical examinations in the Health Management Centre of Tianjin First Central Hospital, China from May 2015 to September 2016. We adhered to the principles of the Declaration of Helsinki, and the study protocol was approved by the Ethics Committee of Tianjin First Central Hospital. All participants gave informed written consent. A total of 450 elderly subjects (age ≥ 65 years) were enrolled our study and finished a comprehensive medical examinations and completed the questionnaire.

### Inclusion criteria

People over the age of 65 that had undergone a general medical examination and baPWV examination in the Health Management Centre of Tianjin First Central Hospital who could walk by themselves without using a walking aid during gait speed measuremen and who did not have a history of mental illness were selected for inclusion in this study. All subjects agreed to participate in the study, and signed informed consent forms.

### Exclusion criteria

Subjects with cognitive impairment or dementia, neurological diseases (central nervous system and peripheral nervous system diseases such as serious cerebral stroke, brain and spinal cord tumors, trauma, inflammation, or Parkinson’s disease), or mental disorders affecting activity; organ failure (heart, lung, liver, kidney); edema, severe endocrine diseases that not be well-controlled; autoimmune diseases; inflammatory disease; malignancy or anorexia; malabsorption or gastrointestinal disorders, and trauma or fracture leading to bed rest were excluded from this study.

### Diagnosis of sarcopenia

Sarcopenia was defined according to the Consensus Report produced by AWGS [1], which is criterion1 and/or criterion2 plus criterion3. Criterion1 was low muscle strength: HS < 26 kg for men and < 18 kg for women. Criterion2 was low physical performance: usual GS < 0.8 m/s; Criterion3 was low muscle mass: ASMI < 7.0 kg/m^2^ for men and 5.7 kg/m^2^ for women as measured by bioimpedance analysis.

### Muscle strength, mass and gait speed

we used the protocol of Roberts et al. for the measurement of grip strength [8]. The participant was asked to sit comfortably on a standard chair with legs, back support and fixed arms. The devices were calibrated prior the measurement of grip strength. The participant was advised to squeeze the Jamar hand dynamometer 5030 J1.

(Lafayette Instrument Company, USA) for up to 6 sec as hard as possible and then relax. Three measurements for each hand alternating sides were performed without rest. The best of the six grip strength measurements was recorded and used in statistical analyses.

GS was determined based on a subject’s usual walking pace over a distance of 6 m in seconds, counted after a one meter initial distance. GS measurement was conducted in a flat and unobstructed clinic hallway.

ASMI was defined as appendicular skeletal muscle mass (ASM)/height(m^2^). ASM and VFA was measured by bioelectrical impedance analysis (InBodyS10, InBody Japan Inc., Tokyo, Japan). Lean body mass as measured by bioelectrical impedance analysis was highly associated with the data evaluated by dual-energy X-ray absorptiometry [9]. But it has been recognised that BIA overestimate muscle mass. Therefore we used a validation equation against DXA in order to get the appendicular lean mass value by BIA closed to that measured by DXA [10]:

$$ \left(\mathrm{DXA}\right)=0.04\ast \mathrm{BMI}\hbox{-} 0.58\ast \mathrm{Women}+0.69\ast \mathrm{ASM}/\mathrm{height}\;\left(\mathrm{m}2\right)\;\left(\mathrm{BIA}\right) $$

### Medical history and living habits

Acquisition of medical history and living habits was conducted using a questionnaire survey that included questions pertaining to smoking habits, sports participation, and past medical history. Smoking was defined as people who have smoked for six consecutive months or six cumulative months. Sports participation was defined as > 30 min in a given session, > 3 times a week and lasting for more than 3 months.

### Body measurements

Measurements of height, weight, and blood pressure (mmHg) were routinely collected, and BMI was calculated.

BaPWV measurements:

On the day of medical examination between 8:00–11:00 a.m., the baPWV was measured via VP1000 (an automatic atherosclerosis tester produced by the C0lin Company of Japan). The room temperature was kept at 22 °C–25 °C. Before measurement, the subjects were instructed not to smoke and to rest for more than 5 min. The subjects were kept quiet, lying on the bed with their hands on both sides of their body. Blood pressure cuffs were tied to the upper arms and lower limbs separatelys. The upper arm cuff was oriented towards the brachial artery and the lower edge of the cuff was 2-3 cm away from the elbow. The lower limb cuff was located at the preaxial portion of that lower limb. The lower edge of the cuff was 1-2 cm away from the medial malleolus. The heart sound acquisition device was placed in the anterior cardiac region of the subject. The left and right wrists were clamped using an electrocardiogram acquisition device. Measurements were repeated twice for each subject, with the second measurement acquired as the final result. In this study, the larger values of baPWV on the left and right sides were analyzed. According to American College of Cardiology medical/scientific report (1993) criteria: arterial stiffness was defined as baPWV> 1400 cm/s.

### Nutrition risk

To evaluate the nutritional risk, subjects were administered the Mini-nutritional Assessment short-form (MNA-SF) [11]; a score of less than or equal to 11 points indicated a risk of malnutrition.

### Laboratory examinations

5 ml of elbow venous blood was drawn in the morning from each subject following a minimum of an 8 h fasting period. This sample was then use to aquire a complete blood cell count, and to measure hemoglobin (Hb) and blood biochemistry indicators including serum total protein, serum ALB, TG, total cholesterol, low-density lipoprotein cholesterol (LDL-C), high density lipoprotein cholesterol, serum Cr, urea nitrogen, blood glucose, and hemoglobin A1C (HbA1C) were tested immediately.

### Statistical methods

All data were analyzed using Statistical software Package for Social Sciences version 19.0 (SPSS 19.0). Continuous variables were given as mean ± standard deviation (SD). Numeration data were described as percentages (%). Continuous variables were Checked for the normality, then non-normal distribution variables were transformed to normal distribution prior running parametric statistics. The differences in means and proportions between the two groups were analyzed using independent samples t tests and chi-squared tests respectively. Multiple linear regression analysis was used to analyze the relationship between baPWV and components of sarcopenia. ASMI, muscle strength and physical performance as components of sarcopenia were tested separately and peformed a final analysis. BaPWV was taken as the dependent variable, ASMI, HS and GS were tested simultaneously as independent variables and models were adjusted for sex, age, BMI, VFA, hypertension, diabetes, cardiac, smoking, sports participation, MNA-SF, TG, LDL-C, HbA1C, Hb, ALB and Cr. Multiple logistic regression analysis was performed to estimate the odds ratios (OR) and 95% confidence intervals (CI) for arterial stiffness in relation to sarcopenia. To understand whether potential confounders could affect OR, we used a multivariate model with adjustment for the following covariates: age, sex, BMI, VFA, hypertension, diabetes, cardiovascular disease, smoking behaviors, physical activity, nutritional risk, TG, LDL-C, HbA1C, Hb, ALB, Cr. A *p* value < 0.05 was considered statistically significant.

## Results

1. A total of 450 elderly subjects (266 male and 184 female, age ≥ 65 years) meeting the inclusion criteria were enrolled our study and finished the program. 89 of these individuals were diagnosed as sarcopenia according to the criteria of AWGS. These subjects included 50 males and 39 females between the ages of 65–90 (mean age 72.48 ± 4.65 years). The other remaining 361 individuals without sarcopenia including 216 males and 145 females between the ages of 65–89 (mean age 71.05 ± 4.15 years) served as the non-sarcopenia group in this study. The general characteristics and the other clinical or biochemical indicators of the participants are listed in Table 1. Subjects with sarcopenia had higher baPWV, nutrition risk and lower ASMI, HS, GS, BMI, TG, serum ALB, and serum Cr than those without sarcopenia. Subjects with sarcopenia also had a higher VFA than did the subjects without sarcopenia. These differences between the two groups were significant (*P* < 0.05). Women compared with men had lower HS (24.08 ± 4.38 VS 26.08 ± 5.50, t = 4.105 *p* = 0.000), GS (1.06 ± 0.3VS 1.15 ± 0.51, t = 2.135 *p* = 0.033), ASMI (7.33 ± 0.74 VS 7.49 ± 0.85, t = 2.051 *p* = 0.041) and BaPWV (1643.94 ± 137.64 VS 1670.59 ± 132.91, t = 2.061 *p* = 0.040) (data not shown in table).

(2) The results of a multivariable linear regression analysis between baPWV and ASMI,muscle strength,physical performance are listed in Table 2. ASMI and HS were negatively associated with baPWV (t = − 5.807, *p* = 0.000 and t = − 3.085, *p* = 0.002), but the relationship between baPWV and GS was not statistically significant (t = − 0.862, *p* = 0.389) by multivariable linear regression. In further analyses by sex, the relationship of baPWV and ASMI, HS still had statistical significance both in men and women; the relationship between baPWV and GS was not statistically significant.

(3) The relationship between arterial stiffness and sarcopenia.

**Table 1:** The general characteristics and clinical data of study participants with and without sarcopenia

Variable	Sarcopenia *n* = 89	Non-Sarcopenia *n* = 361	*x*^2^/*t*	p
Sex (male/female)	50/39	216/145	0.394	0.530
Age (years)	72.48 ± 4.65	71.05 ± 4.15	2.854	0.005
BMI (kg/m^2^)	24.01 ± 1.92	25.20 ± 2.31	−5.030	0.000
VFA (cm^2^)	98.03 ± 13.11	90.71 ± 16.87	4.438	0.000
Hypertension (%)	28 (31.46)	105 (29.09)	0.193	0.660
Diabetes (%)	14 (15.73)	49 (13.57)	0.276	0.599
Cardiac disease(%)	30 (33.71)	101 (27.98)	1.136	0.287
Smoking (%)	15 (16.85)	49 (13.57)	0.630	0.427
Sports (%)	10 (11.24)	75 (20.78)	4.241	0.039
MNA-SF (%)	36 (39.02)	87 (24.10)	9.609	0.002
TG (mmol/L)	1.31 ± 0.45	1.51 ± 0.58	−2.981	0.003
LDL-C(mmol/L)	3.12 ± 0.61	3.08 ± 0.62	0.646	0.519
HbA1C(%)	5.37 ± 0.93	5.28 ± 1.12	0.781	0.436
Hb(g/L)	125.73 ± 11.97	124.98 ± 15.44	0.426	0.670
ALB(g/L)	41.16 ± 4.49	44.19 ± 4.43	−5.769	0.000
Cr(umol/L)	66.12 ± 14.15	72.37 ± 11.15	−4.477	0.000
baPWV (cm/s)	1792.39 ± 128.27	1647.57 ± 121.04	9.989	0.000
HS (kg)	23.99 ± 5.60	25.48 ± 5.72	−2.216	0.027
GS(m/s)	0.86 ± 0.26	1.20 ± 0.58	−5.433	0.000
ASMI (kg/m^2^)	6.59 ± 0.73	7.64 ± 0.76	− 11.738	0.000

*BMI* body mass index, *ALB* serum albumin, *TG* triacylglycerol, *Cr* creatinine, *VFA* visceral fat tissue, *MNA-SF* mini-nutritional assessment short-form, *BIA* Bioelectrical impedance analysis, *ASMI* appendicular skeletal muscle index, *Hb* hemoglobin, *HbA1C* hemoglobin A1C, *LDL-C* low-density lipoprotein cholesterol, *baPWV* brachial ankle pulse wave velocity, *HS* Handgrip strength, *GS* usual gait speed

**Table 2:** Multivariable linear regression analysis between baPWV and ASMI, muscle strength, and physical performance

Variable	Men				Women				All			
	β	95%CI	t	p	β	95%CI	t	p	β	95%CI	t	p
ASMI (kg/m^2^)	−32.752	−48.763 to −23.364	−5.722	0.000	−30.653	−44.576 to − 25.435	−4.325	0.000	−39.783	− 54.861 to − 28.253	−5.807	0.000
HS (kg)	−6.132	−10.153 to − 2.827	−2.863	0.033	−6.127	− 10.124 to − 2.738	−2.085	0.043	−8.007	−13.108 to − 2.907	−3.085	0.002
GS(m/s)	−7.526	−26.231 to 9.368	−0.675	0.562	− 10.273	−25.361 to 9.235	−0.562	0.145	−9.273	−30.413 to 11.868	−0.862	0.389

* baPWV was taken as the dependent variab, ASMI, HS and GS were tested simultaneously as independent variables, and models were adjusted for sex, age, BMI, VFA, hypertension, diabetes, cardiac, smoking, sports, MNA-SF, TG, LDL-C, HbA1C, Hb, ALB and Cr

Univariate analyses were performed investigating the relationship between sarcopenia and baPWV, the other clinical or biochemical parameters measured in this study. The result are presented in Table 3. The associations were observed between sarcopenia and age, BMI, Sport, MNA-SF, VFA and baPWV. Finally, we performed multivariate logistic regression analyses to assess the relationship between sarcopenia and baPWV (Table 4). Following adjustment for potential confounders (age, BMI, sport, MNA-SF and VFA), sarcopenia was related with age, BMI, sports and baPWV, baPWV was a risk factor of sarcopenia (OR = 1.68, 95%CI 1.45–1.87). In further analyses by sex, baPWV was still a risk factor of sarcopenia.

**Table 3:** Univariate regression analysis for correlative factor of sarcopenia

Variable	Men		Women		All	
	OR (CI 95%)	p	OR (CI 95%)	p	OR (CI 95%)	p
Age (years)	1.16 (1.12–1.44)	0.016	1.12 (1.06–1.32)	0.012	1.13 (1.07–1.25)	0.015
male/female(0,1)	–	–	–	–	0.76 (0.65–1.23)	0.375
BMI (kg/m^2^)	0.82 (0.62–0.96)	0.038	0.74 (0.61–0.89)	0.032	0.78 (0.66–0.92)	0.036
Hypertension (0,1)	1.38 (0.78–1.68)	0.068	1.27 (0.91–1.53)	0.072	1.26 (0.87–1.49)	0.052
Diabetes (0,1)	1.72 (0.93–1.82)	0.132	1.43 (0.89–1.66)	0.118	1.58 (0.92–1.87)	0.129
Smoking (0,1)	1.53 (0.85–1.29)	0.324	1.23 (0.79–1.25)	0.221	1.07 (0.86–1.27)	0.238
Cardiac disease(0,1)	0.91 (0.89–1.21)	0.368	0.87 (0.82–1.21)	0.283	0.89 (0.90–1.15)	0.284
Sports(0,1)	0.78 (0.63–0.91)	0.036	0.80 (0.67–0.95)	0.043	0.80 (0.66–0.93)	0.042
MNA-SF(0,1)	0.73 (0.71–0.92)	0.046	0.81 (0.77–0.92)	0.031	0.83 (0.75–0.94)	0.038
TG (mmol/L)	0.82 (0.74–1.03)	0.669	0.89 (0.73–1.12)	0.725	0.88 (0.76–1.08)	0.687
LDL-C (mmol/L)	0.85 (0.65–1.12)	0.628	0.88 (0.67–1.23)	0.792	0.81 (0.47–1.07)	0.531
HbA1C(%)	0.89 (0.81–1.05)	0.488	0.93 (0.82–1.26)	0.631	0.92 (0.81–1.07)	0.512
Hb(g/L)	0.69 (0.65–1.21)	0.081	0.72 (0.65–1.27)	0.124	0.68 (0.61–1.17)	0.074
ALB(g/L)	0.88 (0.79–1.11)	0.058	0.82 (0.75–1.23)	0.051	0.85 (0.76–1.12)	0.052
Cr(umol/L)	0.88 (0.72–1.43)	0.076	0.84 (0.68–1.23)	0.061	0.86 (0.70–1.25)	0.063
VFA (cm^2^)	0.58 (0.58–0.79)	0.039	0.61 (0.47–0.88)	0.043	0.59 (0.48–0.83)	0.041
BaPWV (cm/s)	1.15 (1.05–1.63)	0.019	1.32 (1.09–1.52)	0.036	1.28 (1.07–1.46)	0.027

*OR* odds ratio, *CI* confidence interval, *BMI* body mass index, *ALB* serum albumin, *TG* triacylglycerol, *Cr* creatinine, *VFA* visceral fat tissue, *MNA-SF* mini-nutritional assessment short-form, *BIA* Bioelectrical impedance analysis, *ASMI* appendicular skeletal muscle index, *Hb* hemoglobin, *HbA1C* hemoglobin A1C, *LDL-C* low-density lipoprotein cholesterol, *baPWV* brachial ankle pulse wave velocity, *HS* Handgrip strength, *GS* usual gait speed

**Table 4:** Multiple logistic regression analysis for correlative factor of sarcopenia

Variable	Men		Women		All	
	OR (CI 95%)	p	OR (CI 95%)	p	OR (CI 95%)	p
Age (years)	1.24 (1.08–1.42)	0.032	1.18 (1.05–1.29)	0.025	1.21 (1.07–1.32)	0.026
BMI (kg/m^2^)	0.76 (0.67–0.92)	0.037	0.72 (0.64–0.79)	0.034	0.74 (0.65–0.86)	0.035
Sports(0,1)	0.65 (0.52–0.76)	0.041	0.72 (0.56–0.81)	0.048	0.68 (0.53–0.79)	0.046
MNA-SF(0,1)	1.15 (0.79–1.37)	0.735	1.06 (0.77–1.29)	0.578	1.08 (0.81–1.24)	0.652
VFA (cm^2^)	0.63 (0.55–1.12)	0.261	0.72 (0.58–1.13)	0.328	0.65 (0.52–1.03)	0.271
BaPWV (cm/s)	1.82 (1.5–1.93)	0.029	1.73 (1.52–1.91)	0.041	1.68 (1.45–1.87)	0.037

*OR* odds ratio, *CI* confidence interval, *BMI* body mass index, *VFA* visceral fat tissue, *baPWV* brachial ankle pulse wave velocity, *MNA-SF* mini-nutritional assessment short-form

## Discussion

The prevalence of sarcopenia in this study was 18.8 and 21.2% in men and women respectively. It is somehow lower than the report conducted in older hospitalized patients [12] using the diagnostic criteria of the European Working Group on Sarcopenia in Older People (EWGSOP) [13]. The main findings of this cross-sectional study were that those with sarcopenia had higher baPWV and lower ASMI, HS and GS than did non-sarcopenia subjects. Subjects with sarcopenia also had higher VFA. Multivariable linear regression analysis revealed ASMI and HS were negatively associated with baPWV, but there was no significient association between GS and baPWV. Whereas baPWV was a risk factor of sarcopenia. Even in further analyses by sex, the relationship of baPWV and ASMI, HS still had statistical significance both in men and women; baPWV was also a risk factor of sarcopenia.

We investigated the relationship between sarcopenia and arterial stiffness in the elderly of the community in China based on the criteria included in the Consensus Report of the Asian Working Group for Sarcopenia. In the present study population, baPWV increased as skeletal muscle mass decreased (t = − 5.807, *p* = 0.000). Consistent with the results of our study, several studies have previously reported the associated between skeletal muscle mass decline and arterial stiffness. A cross sectional study conducted in Japan on healthy middle-aged to elderly men demonstrated that low thigh muscle cross-sectional area corrected by body weight was significantly negatively associated with baPWV and carotid intima-media thickness, even after correction for confounding factors including age, body height, and physical activity [5]. Another cross-sectional survey in Japan of 97 hospitalized elderly postmenopausal women with type 2 diabetes mellitus (average age = 65 years) found that after adjusting for confounding factors such as age, body mass index, blood lipids, systolic blood pressure, and duration of onset of type 2 diabetes mellitus, baPWV increased as relative skeletal muscle mass index decreased [6]. In addition, studies have also shown that there is a association between body composition and arterial calcification in elderly men [14, 15].

These previous studies [5, 6] have just found skeletal muscle mass decline is associated with arterial stiffness, but as important compositions of sarcopenia, muscle strength or physical performance were not involved. Only few previous studies [16, 17] estimated the association between muscle strength or physical performance and arterial stiffness. Objects of the few previous studies were not sarcopenia and there are no consistent conclusions. Watson et al. [16] reported that higher PWV was associated with slower gait speed independent of hypertension and other risk factors only among those with peripheral artery disease (PAD), but not in well functioning cohort. Gonzales reported gait performance was inversely associated with carotid–femoral PWV and carotid artery stiffness index after adjustment for age, body mass index, waist circumference and systolic blood pressure [17]. HS are proved to be associated with all-cause mortality and cardiovascular mortality [18, 19]. Hirotomo Yamanashi et al. reported arterial stiffness was associated with HS in non-hypertensive populations, but not in hypertensive populations [20]. Whereas, a long-term study result showed that there was no relation between HS and arterial stiffness [21]. The present cross-sectional study analysed the relation between all compositions of sarcopenia (including skeletal muscle mass, muscle strength or physical performance) and baPWV as marker of arterial stiffness simultaneously. We revealed ASMI and HS as composition of sarcopenia were negatively associated with baPWV (t = − 5.807, *p* = 0.000 and t = − 3.085, *p* = 0.002), but not GS. The result might mean decreased physical performance may have no effect on arterial stiffness.

Conversely, arterial stiffness may also affect sarcopenia. Arterial stiffness induces the decreasing flow of blood, oxygen, and nutrients to muscle tissue, leading to a loss of muscle mass. The Health ABC study, a large cohort study of elderly individuals, investigated the role of arterial stiffening in sarcopenia. The results showed that arterial stiffening as measured based on carotid-femoral pulse wave velocity was associated with a decline in skeletal muscle mass [22]. This present cross-sectional study revealed sarcopenia was related with advancing baPWV (OR = 1.68, 95%CI 1.45–1.87). We speculate that sarcopenia interacts with arterial stiffness.

The pathophysiological mechanism underlying the association between sarcopenia and arterial stiffness have not been fully identified. Timely screening people at risk for sarcopenia and taking effective measures may help control or delay the progress of sarcopenia and arterial stiffness. Defining blood biomarkers in sarcopenia may help characterize the mechanisms of sarcopenia, allowing for early diagnosis and a personalized follow-up for the prevention and treatment measures [23].

There are some limitations to this present study: (1) The gold standard for evaluating arterial stiffness, carotid to femoral pulse wave velocity (cfPWV), was not used in this study. Even so, baPWV is well associated with cfPWV [24], and the AHA had included baPWV in the recommended criteria for evaluating arterial stiffness. (2) The significant differences in the age ranges between sarcopenic and non-sarcopenic participants was a major limitation of this study. Even though age was considered as a possible confounder in the multivariate models, the two groups were non-homogeneous. That may result in deviations from reality. Age matching in future research will be important. (3) The cross-sectional design is another limitation of this study, as we cannot determine cause-effect associations between sarcopenia and arterial stiffness. Therefore, prospective studies are needed. (4) It has been found that polypharmacy was associated with arterial stiffness [25]. We did not consider polypharmacy as a covariate in this study. (5) The relatively small sample size of our study is also a limitation.

## Conclusion

In summary, as important compositions of sarcopenia-ASMI and HS were negatively associated with baPWV in community dwelling elderly in China; and baPWV was a risk factor of sarcopenia. We revealed the interactive relationship between sarcopenia, compositions of sarcopenia and arterial stiffness.

## Data Availability

The datasets generated and/or analyzed during the current study are not publicly available due to the need to protect patient privacy and the Fund provider requires data secrecy.

## Abbreviations

AWGS: The Asian Working Group for Sarcopenia
HS: Handgrip strength
GS: Gait speed
BMI: Body mass index
ALB: Serum albumin
TG: Triacylglycerol
Cr: Creatinine
VFA: Visceral fat tissue
baPWV: Brachial ankle pulse wave velocity
MNA-SF: Mini-nutritional assessment short-form
BIA: Bioelectrical impedance analysis
ASM: Appendicular skeletal lean mass
ASMI: Appendicular skeletal muscle index
Hb: Hemoglobin
HbA1C: Hemoglobin A1C
LDL-C: Low-density lipoprotein cholesterol
SD: Standard deviation
cfPWV: Carotid to femoral pulse wave velocity
OR: Odds ratios
CI: Confidence intervals
EWGSOP: European Working Group on Sarcopenia in Older People
PAD: Peripheral artery disease
